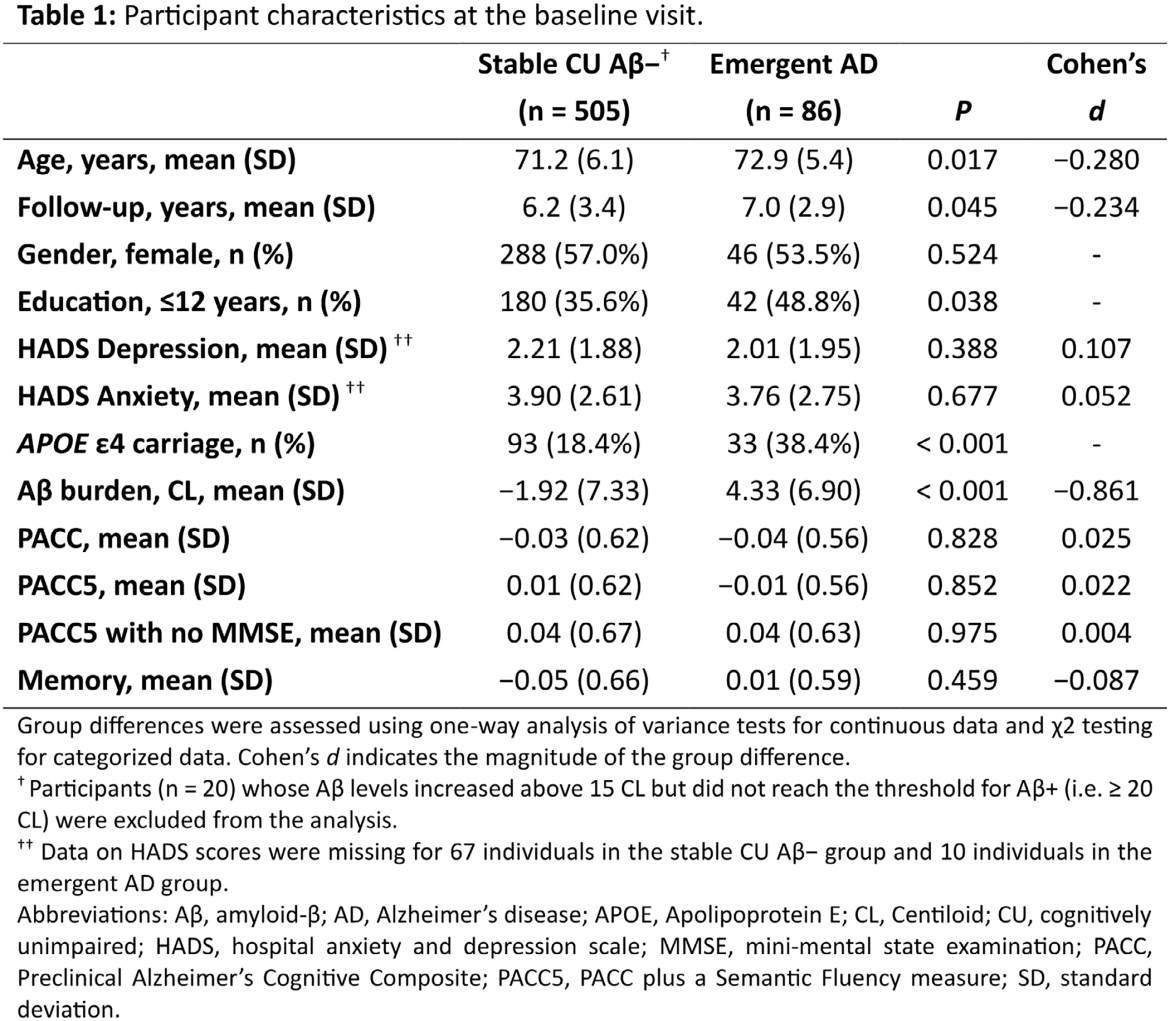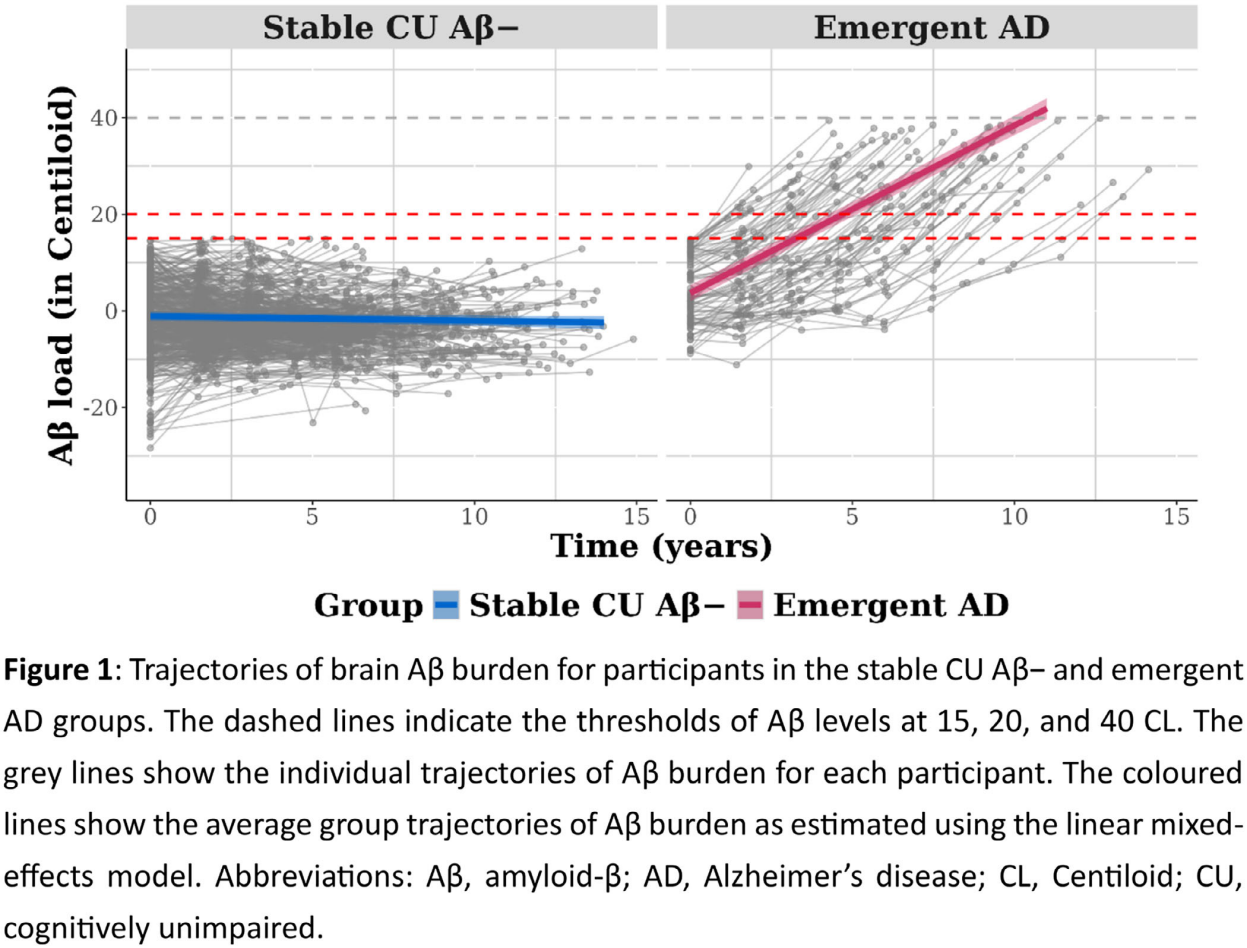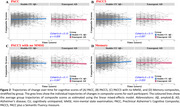# Sensitivity of cognitive composite scores to amyloid accumulation in emergent Alzheimer's disease

**DOI:** 10.1002/alz70856_100567

**Published:** 2025-12-24

**Authors:** Ying Xia, Vincent Dore, Jurgen Fripp, Pierrick Bourgeat, Yen Lim, Joanne Robertson, Christopher C. Rowe, Colin L. Masters, Paul Maruff, for the AIBL study investigators

**Affiliations:** ^1^ CSIRO Health and Biosecurity, Australian E‐Health Research Centre, Brisbane, QLD, Australia; ^2^ School of Biomedical Sciences, The University of Queensland, Brisbane, QLD, Australia; ^3^ CSIRO Health and Biosecurity, Australian E‐Health Research Centre, Parkville, VIC, Australia; ^4^ Austin Health, Heidelberg, VIC, Australia; ^5^ Turner Institute for Brain and Mental Health, Monash University, Melbourne, VIC, Australia; ^6^ The Florey Institute of Neuroscience and Mental Health, The University of Melbourne, Parkville, VIC, Australia; ^7^ Austin Health, Melbourne, VIC, Australia; ^8^ Florey Institute of Neuroscience and Mental Health, University of Melbourne, Melbourne, VIC, Australia; ^9^ Cogstate Ltd., Melbourne, VIC, Australia

## Abstract

**Background:**

In clinical trials for preclinical Alzheimer's disease (AD), cognition is the primary outcome and is measured by preclinical Alzheimer's disease cognitive composite (PACC) scores that combine data from neuropsychological tests of memory and executive function and clinical mental status. As AD prevention strategies target disease stages where amyloid‐β (Aβ) levels are increasing abnormally but remain within normal limits, termed emergent AD, the sensitivity of cognitive measures to disease progression remains unevaluated. This study compared rates of cognitive decline associated with Aβ accumulation in initially Aβ− cognitively unimpaired (CU) individuals, using cognitive composite scores based on memory alone, memory with executive function, and memory, executive function, and mental status.

**Method:**

CU individuals (*n* = 611, age=71.4±6.0 years, 56.5% female, CDR=0) from the Australian Imaging, Biomarkers and Lifestyle (AIBL) study who were initially Aβ− (< 15 Centiloids) underwent repeated Aβ‐PET and cognitive assessments. Four composite scores were computed: the PACC, PACC plus a Semantic Fluency measure (PACC5), PACC5 with no mini‐mental state examination (MMSE), and a memory composite. Rates of cognitive change in these composite scores were compared using linear mixed‐effects models (LMM) between individuals whose Aβ levels increased to meet the criterion for Aβ+ (≥ 20 Centiloids), indicating emergent AD, and those who remained CU Aβ− throughout the study (stable CU Aβ−). In the emergent AD group, follow‐up assessments after Aβ levels exceeded 40 Centiloids were excluded from the analysis.

**Result:**

Emergent AD was classified in 86 of 611 CU participants, while 505 were classified as stable CU Aβ− (Table 1). As anticipated, the emergent AD group exhibited faster Aβ accumulation (β[SE] = 1.225[0.039], *p* < 0.001, Cohen's *d* = 1.63; Figure 1). The LMMs indicated a statistically significant group‐by‐time interaction for PACC, PACC5 (excluding MMSE), and memory composites (Figure 2), with the strongest effect observed for the memory composite (β[SE] = −0.089[0.029], *p* = 0.003, *d* = 0.16).

**Conclusion:**

In individuals with increasing Aβ levels before meeting the criteria for stage 2 AD, the memory composite showed the greatest sensitivity to Aβ‐related cognitive changes. These findings suggest clinical trials for emergent AD should include assessments of memory to capture AD‐related cognitive changes.